# Comparison of Recombinant Human Haptocorrin Expressed in Human Embryonic Kidney Cells and Native Haptocorrin

**DOI:** 10.1371/journal.pone.0037421

**Published:** 2012-05-25

**Authors:** Evelyne Furger, Sergey N. Fedosov, Dorte Launholt Lildballe, Robert Waibel, Roger Schibli, Ebba Nexo, Eliane Fischer

**Affiliations:** 1 Center for Radiopharmaceutical Sciences, Paul Scherrer Institute, Villigen, Switzerland; 2 Department of Engineering, Aarhus University, Aarhus, Denmark; 3 Department of Clinical Biochemistry, Aarhus University Hospital, Aarhus, Denmark; Duke University Medical Center, United States of America

## Abstract

Haptocorrin (HC) is a circulating corrinoid binding protein with unclear function. In contrast to transcobalamin, the other transport protein in blood, HC is heavily glycosylated and binds a variety of cobalamin (Cbl) analogues. HC is present not only in blood but also in various secretions like milk, tears and saliva. No recombinant form of HC has been described so far. We report the expression of recombinant human HC (rhHC) in human embryonic kidney cells. We purified the protein with a yield of 6 mg (90 nmol) per litre of cell culture supernatant. The isolated rhHC behaved as native HC concerning its spectral properties and ability to recognize both Cbl and its baseless analogue cobinamide. Similar to native HC isolated from blood, rhHC bound to the asialoglycoprotein receptor only after removal of terminal sialic acid residues by treatment with neuraminidase. Interestingly, rhHC, that compared to native HC contains four excessive amino acids (…LVPR) at the C-terminus, showed subtle changes in the binding kinetics of Cbl, cobinamide and the fluorescent Cbl conjugate CBC. The recombinant protein has properties very similar to native HC and although showing slightly different ligand binding kinetics, rhHC is valuable for further biochemical and structural studies.

## Introduction

Vitamin B12 (cobalamin, Cbl) is a water soluble vitamin which is involved in biosynthetic processes in every living cell. Its transport within the human body is mediated by an elaborate system involving three soluble binding proteins, haptocorrin (HC) (previously referred to as transcobalamin I or R-binder), intrinsic factor (IF) and transcobalamin (TC) as well as their receptors [1].

IF ensures the uptake of Cbl in the intestinal cell, and once Cbl is transferred to the blood, TC is needed in order to transport Cbl into all cells of the body. The role for HC is only partially known [2]. In the upper digestive tract, salivary HC captures dietary Cbl and mediates its transport through the stomach. Subsequently, HC is degraded by pancreatic enzymes in the duodenum, whereupon the released Cbl binds to IF and is transferred to the blood. In the blood, approximately 75% of all Cbl is bound to HC whereas the remaining fraction is bound to TC [3]. Interestingly, HC binds not only Cbl but also so-called Cbl analogues, which are corrinoids without cofactor activity in mammalian cells. Up to 40% of all corrinoids bound to HC in plasma are Cbl analogues [2], [4], [5].

While the presence of IF and TC is restricted to a few compartments in the body, HC is present in both the circulation and in most exocrine secretions like saliva, tears, and human milk [2].

In contrast to TC, HC is heavily glycosylated and has an apparent molecular mass of 60–70 kDa of which approximately 25% are attributed to glycans. Depending on the source of synthesis, different glycoforms of HC have been described [6]. HC that lacks terminal sialic acid is known to be rapidly cleared by the asialoglycoprotein receptor in the liver [7], while for sialylated HC, no receptor has been identified.

Interestingly, some tumour types, including fibrolamellar hepatocellular carcinoma, have been described to express high amounts of HC [8], [9]. In recent studies, this protein has been proposed as a diagnostic marker in breast cancer [10], [11] and fibrolamellar hepatocellular carcinoma [8]. Expression of HC in cancer can reach high levels, and HC has very recently even been proposed as a target molecule for cancer diagnosis or therapy [11], [12].

Both IF and TC have been expressed in various hosts like plants, yeast, and insect cells [13], [14], [15], [16], and their structures in complex with Cbl have been solved by X-ray crystallography [17], [18]

The overall structure of both proteins is similar and consists of an N-terminal α-domain and a C-terminal β-domain connected by a flexible linker. In contrast to IF and TC, neither expression of recombinant HC nor its crystallisation have been reported so far. Thus, the only source of HC for biochemical studies is purified protein from human serum, saliva, or milk, which hinders protein consuming experiments, e.g. crystallographic analysis.

The present work reports expression, purification and characterisation of recombinant human HC (rhHC) in the cell line HEK293. The C-terminal Myc-His-tag was removed using a thrombin-cleavage site, and the protein was further purified to >98% purity. We compare the biochemical properties of rhHC to native human HC isolated from plasma of a cancer patient [19].

## Results

### Purification of rhHC from HEK293 culture supernatant

**Figure 1:**
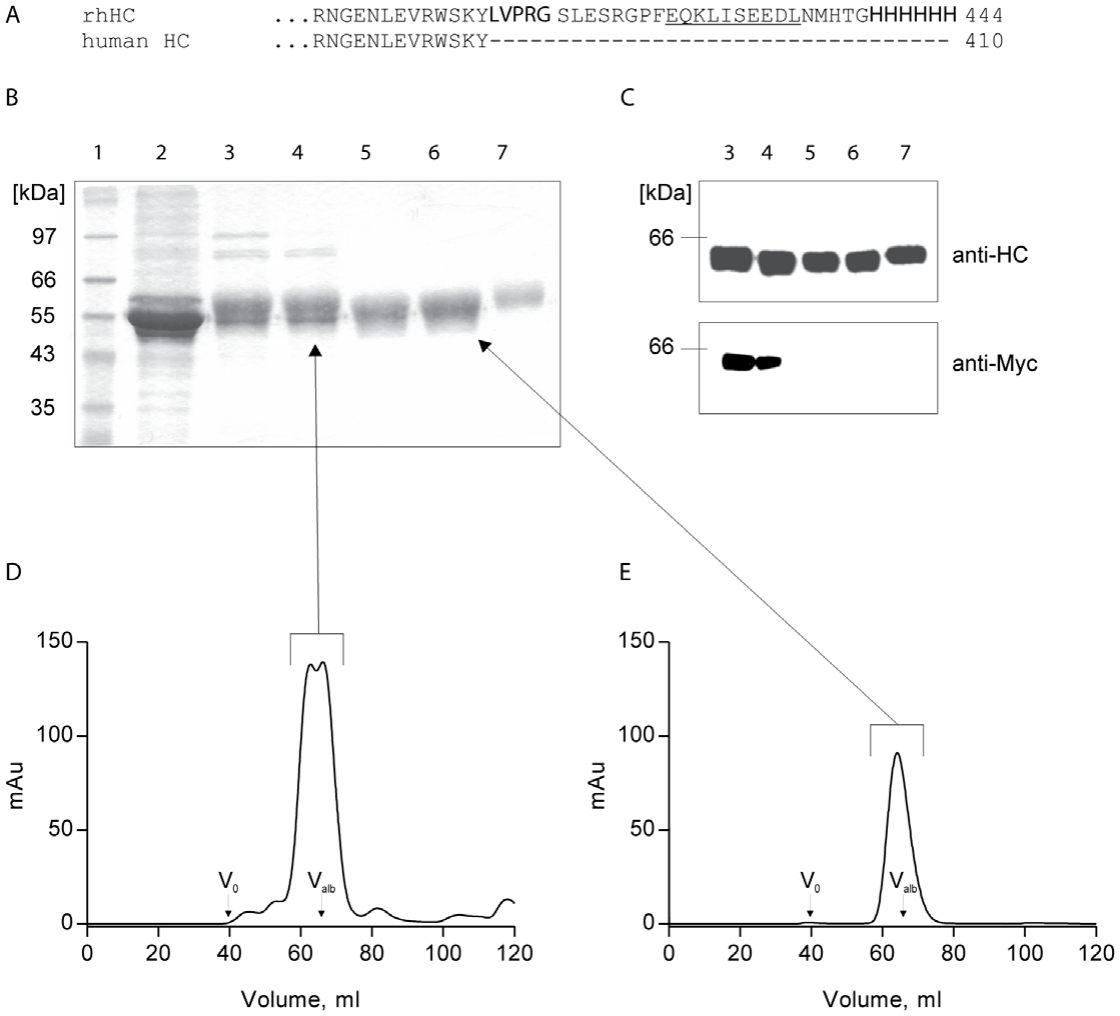
Purification of rhHC. rhHC purified from approximately 0.5 L HEK293 cell culture supernatant. (A) Sequence alignment of native HC and tagged rhHC. Thrombin cleavage site (**bold**), Myc-tag (underlined) and 6×His-tag (*italic*) (B) Coomassie stained SDS/PAGE and (C) Western blot analysis of the different purification steps. Lanes: 1, standards, 2, supernatant of HEK293 cells transfected with rhHC, 3, elution of first Ni^2+^ affinity chromatography (tagged rhHC), 4, peak fraction of first size exclusion chromatography (tagged rhHC), 5, flow-through of second Ni^2+^ affinity chromatography after thrombin cleavage, 6, peak fraction of second size exclusion chromatography, 7, human native HC [9]. (D) and (E) UV-chromatograms at 280 nm of first and second size exclusion chromatography before and after thrombin cleavage on a Superdex 200 column at a flow rate of 48 ml h^−1^. Elution volumes of Blue Dextran 2000 (V_0_) and albumin (V_alb_, 67 kDa) are marked with arrows.

We developed a mammalian cell expression system for production of rhHC, which contained a thrombin-cleavable, C-terminal fusion tag consisting of the Myc and pohlyhistidine peptides (Myc-His-tag) for affinity purification and immunological detection by anti-Myc-tag antibody (Figure 1A). The expressed fusion protein was purified by His-tag Ni^2+^ affinity and size exclusion chromatography. Tagged protein was obtained at high yields (10 mg/L culture supernatant) and relatively high purity (Figure 1B, lanes 3 and 4). Subsequently, the fusion tag was removed from rhHC by cleavage with thrombin and rhHC was further purified by a Ni^2+^ affinity chromatography step to remove uncleaved rhHC, the cleaved tag peptides and any protein impurities with affinity for Ni^2+^ (Figure 1B, lane 5). A final size exclusion chromatography step was performed to remove thrombin (Figure 1B, lane 6). Western blotting of the tagged and untagged products indicated complete removal of the fusion tag after thrombin cleavage (Figure 1C).

Comparison of the two size exclusion chromatograms showed that the tagged intermediate product was eluted as a main rhHC peak with a shoulder and some impurities (Figure 1D), whereas only a single peak was observed in the final untagged rhHC preparation (Figure 1E).

The purified rhHC appeared as one single but broad band on SDS/PAGE after Coomassie staining (purity >98%), and the yield of rhHC was estimated as 6 mg (90 nmol/L) from one litre of cell culture supernatant. N-terminal sequencing (EICEVS…) confirmed the identity and correct maturation of the purified protein. At the C-terminus, four excessive amino acid residues (…LVPR) remained after cleavage with thrombin. The apparent molecular weight of rhHC on SDS/PAGE was comparable to the weight of native HC purified from plasma of a patient hepatocellular carcinoma [20] (Figure 1B and C, lane 7).

### Glycosylation pattern of rhHC

**Figure 2:**
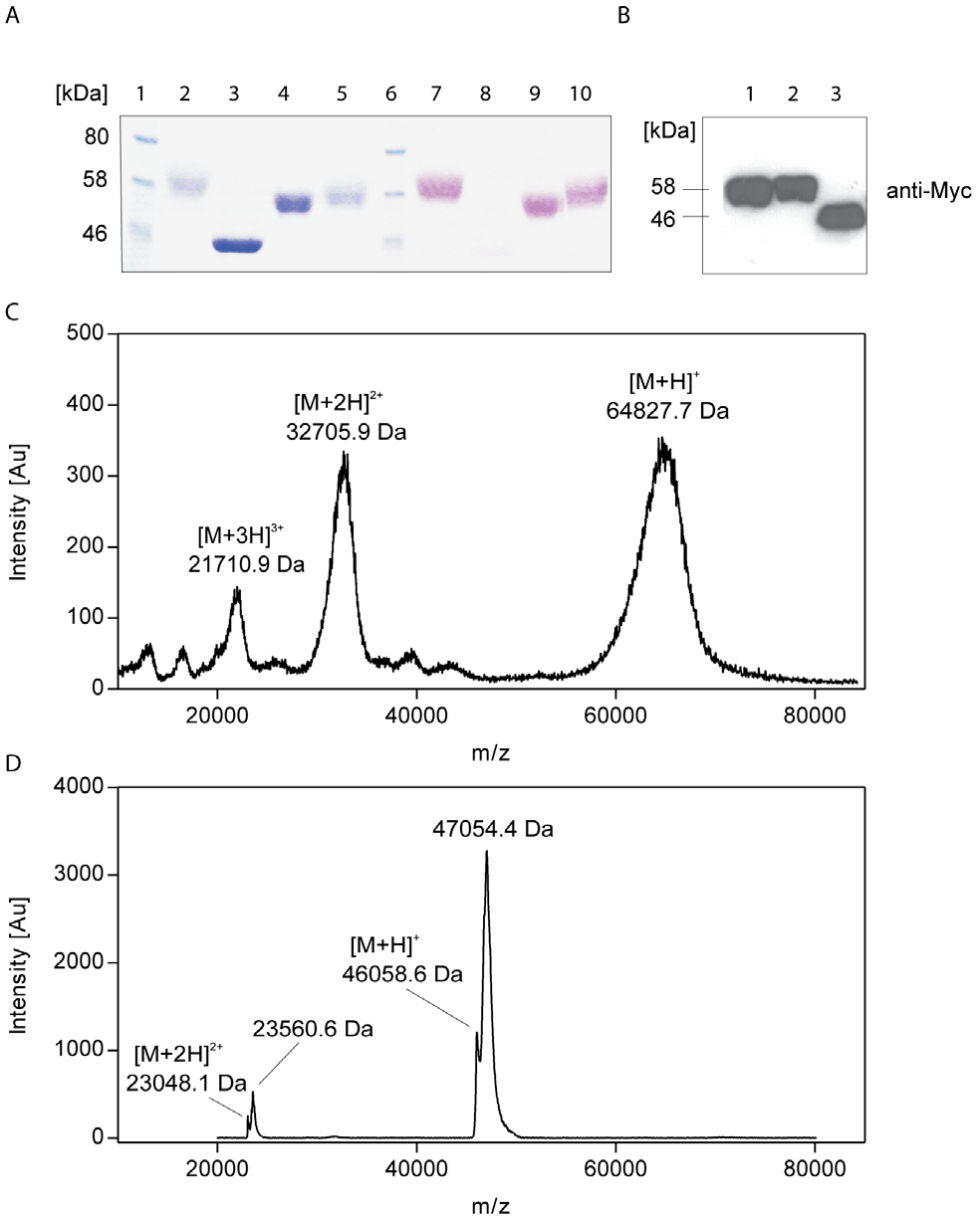
Glycosylation pattern of rhHC. PAS staining revealed heavy glycosylation of rhHC. (A) Coomassie (lane 1–5)/PAS (lanes 6–10) staining of proteins separated on 10% SDS/PAGE. Lanes: 1/6, standards, 2/7, rhHC, 3/8, denatured and deglycosylated (PNGase F) rhHC, 4/9, rhHC overnight incubated with PNGase F and neuraminidase, 5/10, rhHC overnight incubated with PNGase F. (B) Western blot analysis of tagged rhHC using anti-Myc-tag antibody. Lane 1: denatured rhHC, lane 2: rhHC, lane 3: denatured and deglycosylated rhHC. (C) MALDI-MS analysis of rhHC. (D) MALDI-MS analysis of the denatured and deglycosylated (PNGase F) rhHC.

The molecular weight of the purified rhHC was approximately 60 kDa as determined by 10% SDS/PAGE gel stained by Coomassie Brilliant Blue (Figure 2A lane 2) and Western blotting (Figure 2B, lane 1 and 2). This is significantly higher than the theoretical value of the protein backbone of 46 kDa. In agreement with SDS/PAGE, MALDI-MS analysis also revealed a molecular mass of rhHC of approximately 65 kDa (Fig. 2C). The broad peak of mass distribution is expected due to heterogeneous glycosylation patterns of rhHC.

To further analyse the extent of glycosylation, several deglycosylation procedures were used. Incubation with PNGase F alone did not remove N-glycans completely (Figure 2A, lane 5 and 10). Simultaneous treatment of rhHC with neuraminidase and PNGase F resulted in a lower molecular weight of the protein, indicating a high content of terminal sialic acids (Figure 2A, lane 4 and 9). For complete deglycosylation with PNGase F, a denaturation step prior to deglycosylation is needed in order to decrease the protein mass to the expected value of about 46 kDa (Figure 2A, lane 3). MALDI-MS analysis of the denatured and deglycosylated protein showed two peaks: one with the expected mass of the protein backbone 46058.6 Da which was close to the theoretical value of 46076.1 Da for rhHC (NCBI Reference Sequence: NP_001053) including the mass of the four excessive residues present (…LVPR), reduced S-S bonds and assuming eight converted Asn to Asp residues by action of PNGase F [21]. The second peak had a noticeably higher mass of 47054.4 Da (Figure 2D). This strongly indicates remaining posttranslational modification on rhHC even after removal of N-linked glycans. Periodic acid-Schiff (PAS) staining supports this finding, as a residual amount of carbohydrates can still be detected after the deglycosylation procedure (Figure 2A, lane 8).

All together our results indicate that carbohydrates contribute with approximately 20 kDa to the total mass of rhHC.

There are nine sites for N-linked glycosylation showing the consensus sequence Asn-X-Thr/Ser. Database research and previous published studies [22], [23] predict only eight of them to be glycosylated. In contrast to IF with glycosylation only at its C-terminus, the predicted glycosylation sites of HC are situated on both the α- and β-subunit as well as the N-terminal part of the linker region [23].

We analysed the sequence for putative O-glycosylation sites using NetOGlyc 3.1 [24]. Two potential sites for mucin-type O-linked glycosylation were identified on Thr301 and Thr303, which are located on the linker region between the α- and β-subunit of HC. Analysis of the sequences of 25 orthologues (ExPasy) revealed putative O-glycosylation sites in the linker region in 21 of 25 HC-like-sequences (Table S1). Thus, O-glycosylation of the linker may be a common feature of HC-like proteins in various species.

### Absorbance spectra of rhHC

**Figure 3:**
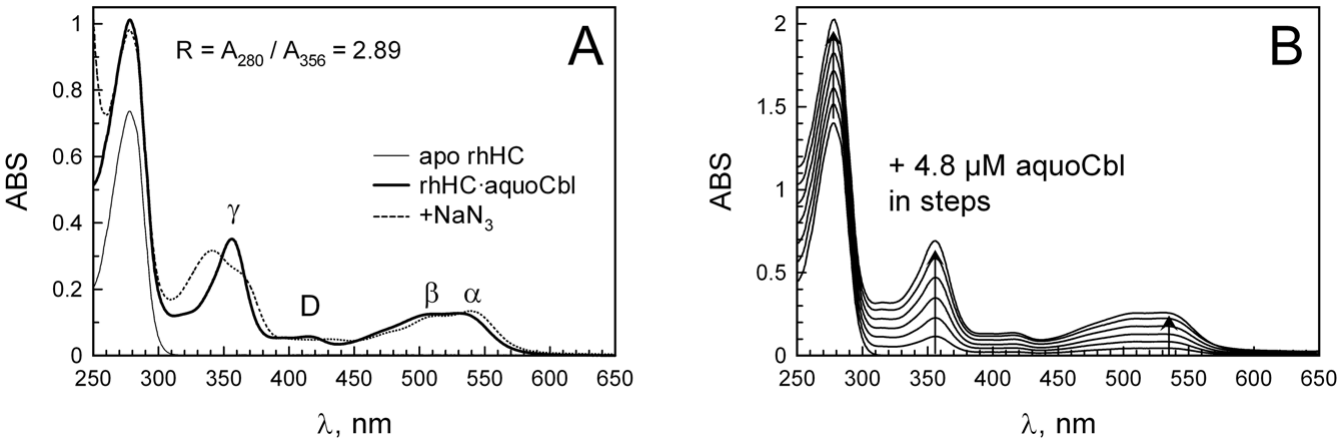
Absorbance spectra of rhHC-Cbl complexes. The absorbance spectrum of Cbl bound to rhHC exhibited a normal transition pattern. (A) Spectrum of rhHC (13.8 µM) in either apo-form or saturated with aquo-Cbl (excess removed) with or without sodium azide (2 mM), pH 7.5, 22°C. (B) Spectra of aquo-Cbl (4.48 µM) added stepwise to rhHC (26.3 µM), dilution was corrected, pH 7.5, 22°C. The data were used for calculation of the coefficients of molar absorbance, see main text and Table 1.

Absorbance spectrum of rhHC in complex with aquo-Cbl is presented in Figure 3A and shows characteristics similar to those previously reported for native human HC (Table 1). Adding azide caused a remarkable shift in the absorption maximum of the gamma peak, which is also characteristic of native human HC [25], [26]. This change is caused by attachment of azide-anion to the “upper surface” of Cbl accompanied by rearrangements in the binding site of HC [26].

**Table 1:** Coefficients of molar absorbance of rhHC (free or in complex with aquo-Cbl, pH 7.5).

Protein species, ε	280 nm	γ, 356 nm	D, 415 nm	β, 509 nm	α, 529 nm
rhApoHC, ε_0_	52·200a)	100	20	20	20
rhApoHC + aquo-Cbl, +Δε	+23·100	+26·100	5·480	9·930	10·030
rhHoloHC, ε	75·900	26·200	5·500	9·950	10·050
Native HoloHC, ε	n.d.	28·600b)	5·400b)	10·300b)	10·400b)

a) Calculated from the composition of the mature protein, ε_280_ = 5500•*N_Trp_* +1490•*N_Tyr_* +125•*N_S-S_* , M^−1^cm^−1^ [42].

b) From Reference [31].

n.d. not determined.

The spectral features of the complex rhHC•aquo-Cbl were explored further when rhHC was saturated with aquo-Cbl added in steps at concentrations below the total binding capacity of the protein (Figure 3B). This allowed calculation of the molar absorbance coefficients of aquo-Cbl when bound to rhHC (Table 1). These parameters helped to evaluate the active protein fraction (AP) as previously shown for IF [27]. For rhHC the value of AP was found from the ratio of R = A_280_/A_356_ according to the equation below and the values of absorbance coefficients from Table 1.
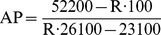



All examined samples of rhHC showed an AP≥0.95 (i.e. ≥95% of the protein bound Cbl stronger than adsorbent of aquo-Cbl, see methods). The best measured values were R = 2.89 and AP = 0.99 indicate an excellent binding activity. In contrast to previously reported purification methods [28], [29], [30], rhHC was produced and isolated directly in the unsaturated (apo-) form without exposure to Cbl-containing adsorbents and denaturing agents. Absence of refolding was apparently beneficial for the protein quality, resulting in a high AP-value. Nevertheless, the effect of GdnHCl treatment was examined on a preparation with AP = 0.95. rhHC was saturated with Cbl and then subjected to dialysis against GdnHCl, see ref. [31] for details. No decrease in the binding activity was found after renaturing of apo-rhHC (AP = 0.95).

### Binding of Cbl and cobinamide to rhHC

**Figure 4:**
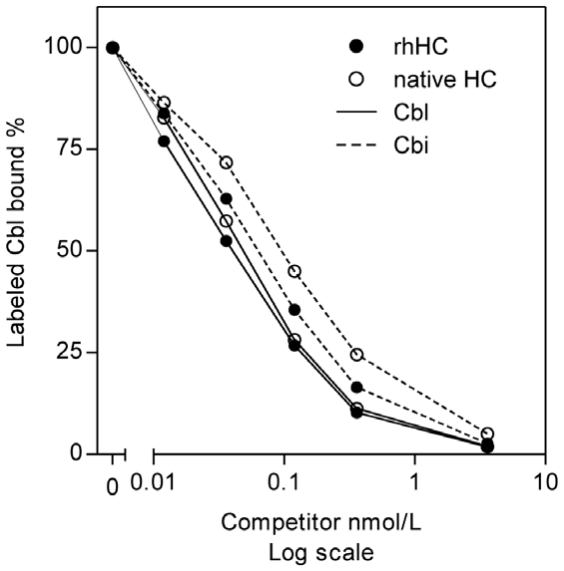
Interaction of rhHC with labelled Cbl in competition with Cbl or Cbi. rhHC and native HC showed a very similar binding behaviour towards Cbl and Cbi. rhHC and native HC were incubated with increasing concentrations of unlabeled Cbl or Cbi (competitor) and the amount of labelled Cbl bound to the proteins was recorded in % of the amount of labelled Cbl bound without the presence of competitor.

The most prominent characteristic of HC is its ability to bind not only Cbl but also Cbl analogues, for example the baseless corrinoid cobinamide (Cbi). We analysed the ability of Cbl and Cbi to compete with labelled ^57^Co-Cbl for the binding to rhHC. Comparable binding curves for rhHC and native HC (Figure 4) point to preservation of this characteristic in rhHC. It should be mentioned, that half-inhibition concentrations, IC50, cannot be connected directly to the dissociation constants (see below). For Cbi this is because equilibration of the mixture of labeled Cbl + Cbi + HC may require several days of incubation.

### Binding and dissociation kinetics of the fluorescent Cbl-conjugate CBC, Cbl and Cbi

**Figure 5:**
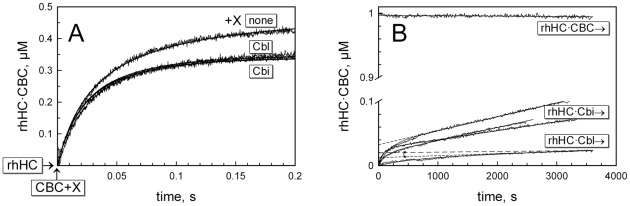
Binding and dissociation kinetics of the fluorescent conjugate CBC and Cbi. Kinetic measurements revealed a subtle difference between rhHC and native HC. (A) Binding kinetics. rhHC was mixed with either CBC or CBC + Cbl/Cbi (all 0.5 µM), pH 7.5, 22°C. Appearance of rhHC·CBC complex was monitored over time according to increasing fluorescence normalized to the maximal amplitude of the signal. The binding rate constants were calculated by computer fitting (solid lines). (B) Dissociation kinetics. rhHC was mixed with either CBC or the non-fluorescent test ligands Cbl and Cbi (all 1 µM, pH 7.5, 22°C, 2 min incubation), whereupon either Cbl (1 µM) or CBC (1 µM) was added. Change in the concentration of rhHC·CBC complex was monitored over time according to the normalized fluorescent response. Dissociation of Cbi was tested in three different preparations of rhHC (all three curves are presented, see also Table 2). Best fits are shown by solid lines. The dissociation rate constants were calculated from the linear slopes of the produced charts (examples indicated by dashed lines). Alternative linear slopes are shown for rhHC·Cbl dissociation.

A more detailed examination of the binding kinetics was performed in a competitive assay, where the fluorescent ligand CBC [32] alone or in the mixture with a test ligand (Cbl or Cbi) reacted with rhHC over time. Attachment of CBC to Cbl-binding proteins causes increase in the quantum yield of the fluorophore, which facilitates monitoring of the protein - CBC interactions [32], [33]. Presence of a non-fluorescent ligand X decreases the signal, because the binding goes along two competing routes HC•CBC ← HC → HC·X (reactions behave as irreversible processes within 0.2 s). The records for interaction between rhHC and either CBC alone or CBC+ Cbl/Cbi mixtures are shown in Figure 5A. The binding and dissociation rate constants *k*_+s_ and k_−s_, as well as the equilibrium dissociation constant K_D_ were calculated for all ligands by computer fitting as explained elsewhere [33] and the results are listed in Table 2. Compared to previously reported values for native HC, all current binding rate constants (*k*_+s_) were 2–3 fold lower. This difference could not be explained by possible concentration error (±10%, examined by simulations). Deterioration of ligands was also ruled out because the concurrent tests with two other proteins (TC and IF) revealed the expected values of *k*_+s_ = 60–70 · 10^6^ M^−1^ s^−1^ for both CBC and Cbl [26], not shown.

**Table 2:** Binding and dissociation rate constants (*k*_+/−CBC_, *k*_+/−Cbl_, *k*_+/−Cbi_) and equilibrium dissociation constants (*K*_d_) at 20°C and pH 7.5.

	*k_+s_*	*k_−s_*	*K_d_*
	*(M^−1^s^−1^)*	*(s^−1^)*	*(M)*
**rhHC**			
CBC	62/64/56b) · 10^6^	≈0.5/2/1b) · 10^−6^	≈20 · 10^−15^
Cbl	X/19/X· 10^6^	X/≤3/X· 10^−6^	≤100 · 10^−15^
Cbi	33/21/Xb) · 10^6^	1.4/2.2/2.1b) · 10^−5^	1000 · 10^−15^
**Native HC** a)			
CBC	124 · 10^6^	≈5 · 10^−7^	(≈4 · 10^−15^)
Cbl	90 · 10^6^	≈5 · 10^−7^	(≈6 · 10^−15^)
Cbi	73 · 10^6^	≈5 · 10^−7^	≈7 · 10^−15^

a) From Reference [33].

b) Measurements on different rhHC preparations with the active protein fractions of 0.99/0.95/0.95 (the last sample was obtained after GdnHCl refolding of rhHC). If no measurement was conducted, the result is notated as X.

The dissociation kinetics was examined in chase experiments which obeyed (within the examined time scale) one of the below schemes: HC·CBC → HC → HC·X or HC·X → HC → HC·CBC (first step was rate limiting). The dissociation kinetics of the ligands CBC, Cbl and Cbi was measured after formation of the corresponding complexes with rhHC, followed by addition of external CBC (or Cbl in the case of CBC itself) (Figure 5B). The dissociation rate constants were then calculated from linear slopes (Table 2). In several cases a small “jump” in the dissociation was observed at the beginning of the reaction (e.g. dissociation curves for Cbl and Cbi, 0–1000 s interval). This fast exponential increase (0.5–3% of the total amplitude) was ascribed to presence of a small fraction of partially denatured protein, where the exchange of ligands was facilitated. This part of the curve was ignored, and only the following linear component was used to calculate the dissociation velocity of the ligand (*v*_−_ = *k*_−s_ · [rhHC•S]). It should be understood, that evaluation of rate constants below 3 · 10^−6^ s^−1^ within the examined time scale cannot be precise, and that the records were used for comparison of different ligands rather than for precise quantification of *k*_−s_.

The rates of CBC and Cbl dissociation from rhHC (*k*_−s_≤3 · 10^−6^ s^−1^) were apparently similar to each other and to the constants reported for native HC (*k*_−s_ = 5 · 10^−7^ s^−1^) measured more accurately over a period of 100 h [33]. On the other hand, dissociation of the analogue Cbi from rhHC was noticeably faster (*k_−Cbi_*≈2 · 10^−5^ s^−1^). An earlier study demonstrated nearly equal dissociation velocities for native HC and a group of corrinoids including Cbl and Cbi (*k_−s_* = 5 · 10^−7^ s^−1^) [33]. Three preparations of rhHC (one after GdnHCl refolding) were examined and none of them showed dissociation of Cbi comparable to that of Cbl or CBC (Figure 5B). Repeated records for the same preparation did not reveal much variation (not shown). Though the overall affinity of rhHC for Cbi was formidable (*K_d,Cbi_*≈1 pM), it was noticeably lower than that of native HC (*K_d,Cbi_* = 7 fM). It seems that adjustment of Cbi in the binding site of rhHC is not completely normal. Considering the two other ligands (Cbl and CBC), difference between rhHC and native HC was less obvious and concerned mostly *k*_+s_.

### Interaction of rhHC with the asialoglycoprotein receptor

**Figure 6:**
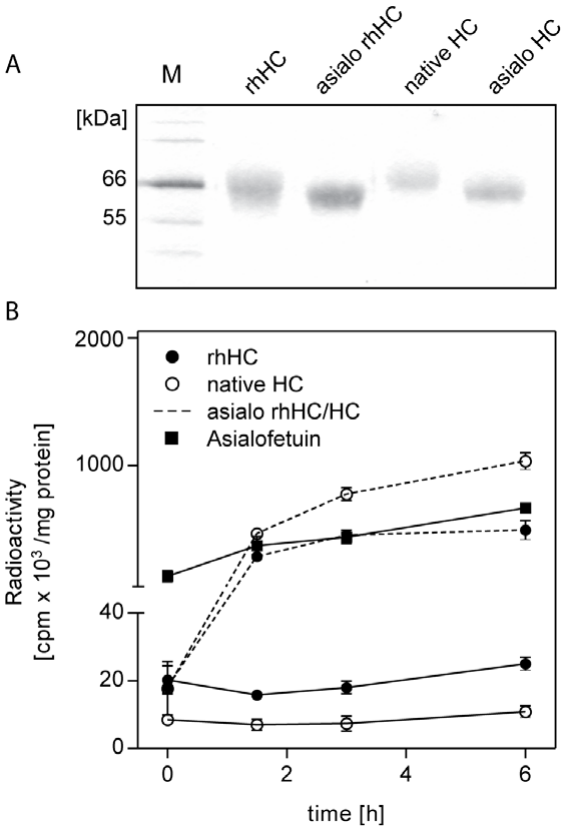
Internalisation of ^125^I-rhHC into HepG2 cells. Comparison of rhHC and human native HC with or without pre-treatment with neuraminidase in order to remove terminal sialic acid. (A) SDS/PAGE analysis after treatment with neuraminidase. (B) Internalisation of ^125^I labelled proteins after 0, 1.5, 3 and 6 h incubation time. Standards are indicated with M.

Depending on the source of expression, different isoforms of HC exist in human blood and extracellular fluids [19]. Notably, different half-lives are associated with these isoforms, probably caused by fast clearance of HC-isoforms with low sialic acid content by the asialoglycoprotein receptor [7]. We tested whether ^125^I-labelled rhHC would be readily taken up by HepG2 cells expressing the asialoglycoprotein receptor involved in HC clearance [34]. As with native HC, rhHC was not internalised over the measured time span of 6 h (Figure 6B). Only after removal of terminal sialic acid by neuraminidase, rhHC and native HC were internalised. Removal of the terminal sialic acid residues was qualitatively determined by SDS/PAGE (Figure 6A). The cellular uptake did not depend on the saturation of the protein with Cbl (Data not shown).

## Discussion

We here report the first successful expression system for recombinant HC. Unlike the expression systems used for production of IF and TC, mammalian cells, such as human embryonic kidney cells, are able to glycosylate proteins in patterns characteristic of higher eukaryotes, yielding products that are expected to be highly similar to their natural human counterparts [35].

In comparison to other sources of human HC such as saliva (15–72 nmol/L), plasma (0.25–84 nmol/L) or milk (4.5–180 nmol/L) [2], the obtained quantity of recombinant protein (90 nmol/L) is comparable or substantially higher. The protein demonstrated a high Cbl binding capacity of 95–99%, and its glycosylation pattern indicated the presence of N- and O-glycosylations. Unlike HC present in saliva and granulocytes rhHC was highly sialylated. Interaction with asialoglycoprotein receptor took only place after removal of terminal sialic acids. We therefore concluded that rhHC represents an isoform that is comparable to HC present in human serum.

We demonstrated the recombinant protein's ability to bind Cbl as well as its baseless analogue Cbi. Furthermore the absorbance spectrum of rhHC showed close similarity to the spectrum of native HC. The spectral transition in rhHC and native HC testifies that the organization of the Cbl binding site in rhHC is comparable to that of native HC.

At the same time, the binding rate constants of all ligands were lower than those of native HC. Dissociation velocity of the examined analogue Cbi was also higher than expected. These subtle differences of rhHC compared to native HC were explained by presence of a peptide elongation attached to Tyr410 involved in the interaction with Cbl.

The finally isolated form of rhHC had four excessive residues (…LVPR) remaining at the C-terminal end discriminating it structurally from native HC. We conjecture that attachment of the excessive peptide (…LVPR) to the C-terminal residue might affect to some degree the binding of ligands resulting in lower affinity. This peptide is directly conjugated to Tyr410 which is possibly involved in the binding of Cbl according to the 3D model of HC-Cbl complex deduced from the crystallographic studies of TC and IF [23]. Such a disturbance might negatively affect the overall affinity *K*_d_ and the binding rate constant *k_+s_*, because the C-terminal β-domain is suggested to be the primary binding site of all corrinoids [27], [36]. Reduction in affinity was particularly visible for the analogue Cbi, whose binding probably requires more structural accuracy of the protein.

In conclusion, we report that rhHC is comparable to native HC and the expression system provides us with the possibility to produce the protein in a sufficient amount for further biochemical and structural studies.

## Materials and Methods

### Materials

Most reagents including salts and buffer components were analytical grade and obtained from Sigma (Sigma-Aldrich Logistics GmbH, Buchs, Switzerland), unless stated otherwise. Ni-NTA Agarose was purchased from Qiagen (Hombrechtikon, Switzerland). Restriction endonucleases, DNA ligase and *Pfu* DNA Polymerase were obtained from Fermentas (Thermo Fischer, Lausanne Switzerland) For comparative studies we used native human HC present in saliva for binding studies [37] or purified to homogeneity from human plasma as previously described [30] for studies on the purified protein. Saliva was collected as part of a previously published research project on HC in saliva [38] and as stated in the paper all of the individuals gave informed consent. Plasma was obtained from a patient with hepatocellular carcinoma as left-overs from routine testing [9].

### Cell Culture

HEK293 cells (DSMZ-Deutsche Sammlung von Mikroorganismen und Zellkulturen GmbH, Braunschweig, Germany) were grown in Dulbecco's modified Eagle's medium containing 4.5 g/L glucose, supplemented with 10% fetal calf serum, 100 U/mL penicillin, 100 µg/mL streptomycin, 0.25 µg/mL fungizone, and 2 mM glutamine in a 5% CO_2_ incubator at 37°C. HepG2 cells (Institut für angewandte Zellkultur, Dr. Toni Lindl GmbH, Munich, Germany) were grown in minimum essential medium with Earl's basal salts supplemented with 10% fetal calf serum, 100 U/mL penicillin, 100 µg/mL streptomycin, 0.25 µg/mL fungizone, 2 mM glutamine, 1% non-essential amino acids, and 1 mM sodium pyruvate. All tissue culture reagents were purchased from BioConcept (Basel, Switzerland).

### Plasmid Construction

The cDNA for human HC (pCMV6-TCNI RC222285, Myc-DDK-tagged ORF clone of Homo sapiens transcobalamin I (TCN1) NCBI Reference Sequence: NM_001062.2, as transfection-ready DNA) purchased by Origene (Lab Force, Nunningen, Switzerland) was cloned into the EcoRI/XhoI site of pcDNA4/*myc*-His A vector (Invitrogen AG, Basel) by standard molecular biological techniques. A thrombin cleavage recognition site (LeuValProArg↓GlySer) was positioned at the fusion junction to release the HC protein from its tag. The primers (Microsynth AG, Balgach, Switzerland) used were 5′-TAATACGACTCACTATAGGG-3′ and 5′-CCGCTCGAG AGATCCACGCGGAACCAGGTATTTGCTCCAGCGAACC-3′. All selected clones were verified by DNA sequencing (Microsynth AG, Balgach, Switzerland).

### Expression of rhHC

Cells were transfected using Lipofectamine 2000 (Invitrogen AG, Basel) following the manufacturer's instructions. After 72 h, the cells were detached with phosphate-buffered saline (PBS)/1 mM EDTA and replated onto 10 new 10 mm cell culture dishes in complete medium supplemented with 400 µg/mL Zeocin™ selection reagent (Invitrogen AG, Basel, Switzerland) and 20 mM HEPES, which was changed every 4–5 days. After about 3 weeks, resistant colonies were isolated and grown as described elsewhere [39].

### Purification of rhHC and thrombin treatment

High producer transfectomas were stimulated with 6 mM sodium butyrate in growth medium (without Zeocin and HEPES) for 8 days. The cell culture medium was centrifuged for 30 min at 7000 g, 4°C, clarified by filtration through a 0.2 µM Express plus filter (Millipore AG, Zug, Switzerland) and concentrated using an Amicon Filter device (MWCO 10000, Millipore AG). After adding 300 mM NaCl and 5 mM imidazol to the obtained solution (0.2 L) it was applied to a Ni^2+^ affinity column. Absorption of rhHC was carried out at room temperature with gravity flow. The resin was washed with 6 volumes of PBS, 6 volumes of PBS/1 M NaCl and 6 volumes of PBS/0.5 M NaCl/20 mM imidazole. Bound proteins were eluted in 1 mL fractions with PBS/300 mM imidazole. Fractions were analysed by SDS/PAGE, and the peak fractions were polished using HILoad™ 16/60 Superdex 200 gel filtration column and Äkta™*prime* system (Amersham Biosciences). Gel filtration was conducted at room temperature at a flow rate of 48 mL h^−1^ using PBS.

The peak fractions were then dialyzed against thrombin cleavage buffer (20 mM Tris-HCl pH 8, 150 mM NaCl, 2.5 mM CaCl_2_) and incubated for 72 h at room temperature with thrombin (from human plasma, Sigma) (5 U/mg protein) on an orbital shaker. The Myc/His-tag was then removed by using a Ni^2+^ affinity column (2.5 mL) with gravity flow and the flow through was collected in 1 mL fractions using PBS. The rhHC-containing fractions were then subjected again to gel filtration as described above. The fractions with pure and untagged rhHC were pooled and concentrated to 1–2 mL by ultrafiltration on an Amicon filter (MWCO 30000, Millipore AG). The protein was stored frozen at −20°C.

### Edman degradation

The Edman degradation was performed at the Functional Genomics Center Zürich, Switzerland.

5 µl of the sample was diluted with 100 µl 0.1% trifluoroacetic acid (TFA) and loaded on a PVDF membrane (ProSorb, Applied Biosystems). The membrane was washed with 100 µl 0.1% TFA and dried. Edman degradation was performed on a Procise 492 cLC (Applied Biosystems) according to the manufacturer's instructions.

### Deglycosylation of rhHC

rhHC was denatured for 5 min at 95°C in denaturation buffer (100 mM, pH 8 Na-phosphate, 25 mM EDTA, 0.5% (w/v) Triton X-100, 0.2% (w/v) SDS, 1% (v/v) β-mercaptoethanol), cooled down and deglycosylated using the enzymes PNGase F (Roche diagnostics GmbH, Rotkreuz, Switzerland) and neuraminidase (Sigma-Aldrich, Switzerland) according to Morkbak et al. [40].

### SDS/PAGE and Western blot analysis

Proteins were separated by SDS/PAGE under reducing conditions and were transferred to PVDF (polyvinylidene difluoride) membranes (Millipore AG, Switzerland) with a semi-dry blotting device (Bio-Rad Laboratories AG, Reinach, Switzerland). Incubations with anti-Myc-tag mouse mAb (9B11) (Cell Signaling Technology, BioConcept, Basel, Switzerland) or Transcobalamin I (M-16) (Santa Cruz Biotechnology, Lab Force, Nunningen, Switzerland) were according to the manufacturer's protocol overnight at 4°C in TBST (20 mM Tris HCl pH 7.5, 500 mM NaCl, 0.05% Tween 20) containing 2% BSA. Detection was done with secondary horseradish peroxidase (HRP)-linked antibodies from Cell Signaling Technology and Santa Cruz Biotechnology, Inc. and the enhanced chemiluminescence (ECL) western blotting substrate from Pierce (Perbio Science Switzerland S.A., Lausanne, Switzerland).

### Mass spectrometric analysis

The MALDI-MS analyses were performed at the Functional Genomics Center Zürich, Switzerland. The denatured and deglycosylated sample was first precipitated by Trichloroethane (TCA). The dried pellet or 1 µl of the untreated sample was dissolved in 5 µl hexafluoro-2-propanol (HFIP) diluted 1∶10 in matrix (saturated sinapinic acid (CHCA) in 0.1% trifluoroacetic acid (TFA) and 50% acetonitrile) and then spotted on the target. In order to get rid of salts, buffer and anorganic impurities, the dry crystals were then washed with 0.1% TFA solution and air dried. The samples were then analysed by MALDI-MS on a Bruker Autoflex II mass spectrometer equipped by a 337 nm Nitrogen laser.

### Spectral studies

Absorbance spectra were recorded on Varian Cary 50 spectrophotometer (Varian, Australia) at 5–15 µM concentrations of Cbl and rhHC dissolved in 0.2 M Na-phosphate buffer, pH 7.5, 22°C.

Prior to AP measurements, rhHC was saturated with an excess of aquo-Cbl, free aquo-Cbl was bound to the specific tetrazole adsorbent [41], whereupon the spectrum of rhHC·Cbl was recorded and compared to the control sample (buffer + aquo-Cbl + adsorbent). After subtraction of the control spectrum, the absorbance ratio and AP were calculated.

### Binding of Cbl and Cbi to rhHC

The ability of rhHC to bind Cbl (Sigma-Aldrich, Denmark) and Cbi (Sigma-Aldrich, Denmark) was explored using a competitive assay previously described [37]. In brief, rhHC or partly purified native apoHC was incubated with ^57^Co-Cbl (Kem-En-Tec, Taastrup, Denmark) together with increasing amounts of Cbl or Cbi overnight. Unbound ^57^Co-Cbl was removed by charcoal precipitation and bound ^57^Co-Cbl was measured employing a Wizard Automatic Gamma Counter (Perkin Elmer).

### Refolding of rhHC

rhHC (10 µM) was saturated with an excess of CNCbl (20 µM) and subjected to dialysis against a 50-fold volume excess of 5 M GdnHCl (30°C, 180 rpm). The solution was changed at days 2 and 3. Incubation was stopped after day 4, whereupon rhHC was renatured by dialysis against 0.2 M Na-phosphate buffer pH 7.5, see also ref. [31].

### Binding studies with fluorescent Cbl-conjugate CBC

All reactions were performed in 0.2 M Na-phosphate buffer, pH 7.5, 22°C. The binding kinetics was monitored by means of stopped flow method (DX.17 MV stopped-flow spectro-fluorometer, Applied Biophysics, UK) using the fluorescent response from a Cbl-conjugate CBC [32], [33], excitation 525 nm, emission >550 nm, slit 1.5 mm, voltage 390, bandpass of 18.6 nm, light path of 1 cm. The binding reaction was started after mixing of rhHC either with CBC alone or CBC + corrinoid. The final concentrations of all reacting species were 0.5 µM. The maximal amplitude of the fluorescent response in the mixture rhHC + CBC corresponded to 0.5 µM of rhHC•CBC complex. Competition between CBC and a non-fluorescent ligand for the binding to rhHC decreases the amplitude. The relevant rate constants of association were calculated by computer fitting, as described elsewhere [32], [33].

The dissociation reactions were monitored on fluorometer Varian Eclipse (Varian, Australia) using the fluorescent response of CBC, excitation 525 nm (slit 5 nm), emission 550 nm (slit 5 nm), photomultiplier 600 V. In one setup the pre-formed rhHC·CBC complex (1 µM, 2 min) was mixed with free Cbl (1 µM), and decrease in fluorescence was registered due to rhHC·CBC ↔ Cbl exchange. Dissociation of the non-fluorescent ligands (Cbl or Cbi) was measured in similar chase experiments. The test ligand was mixed with rhHC (1+1 µM, 2 min), whereupon 1 µM of exogenous CBC was added. The endogenous ligand dissociated from the protein (rate limiting step), whereupon CBC immediately bound to rhHC and increased fluorescence.

### Internalisation studies

The proteins were labelled with ^125^I using the Iodogen method (Pierce). Labelled protein was purified on a PD10 column (GE healthcare, Glattbrugg, Switzerland). HEPG2 cells were incubated in twelve-well plates in triplicates with about 1 µg of labelled protein for 1 h at 4°C. The plates were then incubated for 0, 1.5, 3, 6 h at 37°C for internalisation. After these times, the cells were washed twice with PBS/0.1% bovine serum albumin and then incubated for 5 min with ice-cold acid wash buffer (50 mM Glycine-HCl, 100 mM NaCl, pH 2.8) to remove the cell-surface bound radioligand. Subsequently, cells were solubilised by incubation with 1 N NaOH for 5 min. at 37°C. At each time point the radioactivity in the cell medium (released), the acid wash (cell-surface bound) and the solubilised cells (internalised) were determined with a NaI-γ-counter (Cobra II Packard, Canberra Packard GmbH, Frankfurt, Germany).

## Supporting Information

Table S1 **Amino acid sequences of the linker region connecting the alpha- and beta-domains of HC aligned from different species. Putative N-glycosylation sites are indicated in red.** Putative O-glycosylation sites (predicted by NetOGlyc 3.1) are indicated in bold letters.
(DOCX)
