# Supplementary material for: Comparison of Recombinant Human Haptocorrin Expressed in Human Embryonic Kidney Cells and Native Haptocorrin
Source: PLoS One. 2012 May 25;7(5):e37421. doi: 10.1371/journal.pone.0037421 (PMC3360681; doi:10.1371/journal.pone.0037421)
Supplement: Table S1 — Amino acid sequences of the linker region connecting the alpha- and beta-domains of HC aligned from different species. Putative N-glycosylation sites are indicated in red. Putative O-glycosylation sites (predicted by NetOGlyc 3.1) are indicated in bold letters. (DOCX) [file pone.0037421.s001.docx]

**Table S1**

**Species Sequence**

ORNAN14853 ALEGKTYLDVTR--GCCDSDP-DNL**T**L**SS**PSP**T**AP**TN**-P**ST**I**T**V**T**YKV**T**DGISNTFADSI

ANOCA05883 SLWGRTYLDVNR--FNCSKDKGDHFTYFAFQQIMPFASQRSITVIY------SNRFSEST

MACEU14399 ALEGKTYLDN----VSCQSTP-GHLSI**T**PKP**T**EP**T**HQFYESISVTYR---VVDSI**N**KTST

TURTR11138 ALMGKTYLDVNK**N**SSCACSPDKF**N**ISTDEPVPV**T**P**T**ISLSNISV**N**YS---VQINEIF**N**ST

LAMPA04138 ALMGKTYLDVNNHSACARSPVDF**N**ISSDDPVSV**T**PTVSPSNI**T**VNYY---VQI**N**ETQP-I

BOVIN15542 ALMGKTYLDVTN-PSCGLNPVKF**N**TSTEKPG**T**V**T**PTTAPLNILVKYS---VRI**N**KTSH-T

CANFA13900 ALLGKTYLDVNKDSPCVYKSGAF**N**LSTQEP**T**SGPPAVSHPQIQV**N**YS---VVI**N**TTYN-I

FELCA05217 ALMGKTYLNVNKDSPCVYGPGDF**N**IS**T**VEPI**T**V**T**PPLSPSQIQV**N**YSV--VIIEETHS-T

**HUMAN26634** ALMGKTFLDINKDSSCVSASGNF**N**ISADEPI**T**V**T**PPDSQSYISV**N**YS---VRI**N**ETYF-T

PANTR07054 ---------------------NF**N**ISADEPI**T**V**T**PPDSQSYISV**N**YS---VRI**N**ETYF-T

PONAB07473 ALMGKTFLDINKDSSCVSASGNF**N**ISSDEPV**T**V**T**PPDSQSYISV**N**YS---VRI**N**ETYF-A

MACMU16369 ALMGKTFLDVNKDSSCVSAAG**N**FS**N**S**T**HEPV**T**V**T**APGSQSSISV**N**YS---VTI**N**ETYS-A

CALJA02898 ALLGKTYLDVNRDSSCVSGSGNF**N**ISIPEPV**T**V**T**PPESQSNISV**N**YS---VRI**N**ETYS-A

SPETR03410 ------------------------------------------------------------

OTOGA09004 AVMGKTYLDVN---SCVSSAGNF**N**ISNPVPI**T**VSPPDSPSNISV**N**YS---VRI**N**ETYF-T

TARSY06067 ALVGKTYLDINNNSACVSGSGIF**N**VSIHEPI**T**M**T**PPVSLSYISV**N**YS---VRISETYS-I

LOXAF05754 ALMGKTYLYVNKDSPCVSGSD-F**N**VSAPVPGSV**T**PENSPSAI**T**VRYS---VQI**N**ETYS-T

ECHTE10303 ----------------------L**N**VSIPE**S**GPV**S**PPPPS**S**LI**T**VHY**S**---VKV**N**KTDS-T

PROCA06728 ALLGKTYLDVNK**N**SSCVYRKD-IKVVTPVSTSVIP**T**KSPSLITVHYS---VQI**N**KTYS-T

CHOHO05486 ALMGKTYLDVNKDAPCVYDN--F**N**ISIHEPGPAPPTKLPSVI**T**VHYS---VQI**N**ETYS-T

SORAR02966 ALLGKTYLDVNKDSSCVYDSGKF**N**VSTHQPLSVTPTHAIADISVHYS---VKI**N**ETYS-T

PTEVA01690 ALMGKTYLDINKNSFCVNGSGNFSISIHEPVSVASSSSSSYISVHYS---VKI**N**ETYP-T

HORSE17505 ALVGKTYLDINKDSPCVSDPD-FNSP**T**PKP**T**SERYTNSPSNILVHYS---VKI**N**RTYS-T

RABIT02686 ALMGKTYLDVSKDSSCVSGSGTF**N**ISSHVPKSV**TT**PNPPSNISV**N**Y**S**---VVI**T**KTYS-T

CAVPO17819 ALVGKTYLDVSQDSSCASRLGNSHISSHKPTSITPLNSPLDTSVHYS---VKIS**N**-YS-T

DIPOR07926 PLLGKTYLDVTKDSPCTYGLGNSPLSIPVR**TT**V**T**PTYSPSLISVHYS---VKI**N**KTYS-I

Amino acid sequences of the linker region connecting the alpha- and beta-domains of HC aligned from different species. Putative N-glycosylation sites are indicated in red. Putative O-glycosylation sites (predicted by NetOGlyc 3.1) are indicated in bold letters.
